# Oesphageal Stenting for palliation of malignant mesothelioma

**DOI:** 10.1186/1749-8090-3-3

**Published:** 2008-01-25

**Authors:** Enoch Akowuah, Joseph Rahamim

**Affiliations:** 1 Department of Cardiothoracic Surgery, Derriford Hospital, Plymouth NHS Trust, Plymouth, Devon, UK

## Abstract

Dyspahgia in patients with malignant mesothelioma is usually due to direct infiltration of the eosophagus by the tumour. It can be distressing for the patient and challenging for the physician to treat. We describe three cases in which this condition has been successfully palliated with self expanding esophageal stents.

## Introduction

The incidence of malignant pleural mesothelioma (MM) is rising rapidly in many countries and it continues to be a challenging clinical problem. Dysphagia complicating the disease is due to direct esophageal extension of the tumor and has been infrequently described [1-3]. In these cases, dysphagia has been a late complication and usually a terminal event. There is only one reported case of attempted surgical palliation with an endo-oesophageal tube placed under general anesthetic via a gastrotomy [4].

Over recent years, we have acquired extensive experience in the use of endoluminal self expanding metal stents (SEMS) via endoscopy of the oesophagus for palliation of esophageal malignancy and for benign strictures. In this report, we describe a series of patients with MM presenting with dyspahgia and treated by esophageal SEMS.

## Case presentation

### Patient 1

A 79 year old gentleman with a history of previous asbestos exposure presented with left sided pleural effusion in February 2000. He underwent VATS drainage of the effusion and a pleural biopsy which confirmed a diagnosis of MM (epitheliod type). He was treated by radiotherapy and remained well till July 2001 when he presented with dysphagia. CT confirmed esophageal extension of the tumour and at endoscopy narrowing of the esophagus from 15 to 30 cm was observed. A 15 cm SEM was inserted. The SEM was deployed in a satisfactory position but only had a slit like opening therefore we decided to perform balloon dilatation within the stent.

Balloon dilatation was performed under fluoroscopic guidance. A 50 mm balloon was positioned across the narrowed region of the stent. The balloon was inflated up to 3 atmospheres pressure until a satisfactory lumen within the stent was observed. A water soluble contrast swallow was performed after the procedure.

The patient was discharged and was well for 4 months before presenting again with dysphagia and weight loss. At endoscopy, the previous stent was patent, however a new section of oesophagus distal to the stent was narrowed. This was dilated with to 17 mm with Savary-Gillard dilators and a new SEMS placed from inside the original stent extending into the stomach. He was discharged from hospital but died 8 months later without any further dysphagia.

### Patient 2

A 67 year old gentleman, with a history of previous asbestos exposure, presented with weight loss dyspnoea and left sided chest pain in September 2002. Chest x-ray showed a left sided pleural effusion. CT confirmed the effusion and pleural thickening extending into the mediastinum and revealed para cardiac lymph nodes. In December 2002, He had a bronchoscopy and VATS drainage of the effusion and talc pleurodesis. Pleural fluid cytology and pleural biopsies confirmed sarcomatoid MM.

He was treated with radiotherapy to the chest wall and remained well until June 2003 when he presented with progressive dysphagia. CT showed tumour invading the esophagus and endoscopy showed extrinsic compression of the oesophagus from 30 to 40 cm. A 10 cm SEM was inserted. He survived a further 6 months with good level of activity and no stent related complications.

### Patient 3

**Figure 1:**
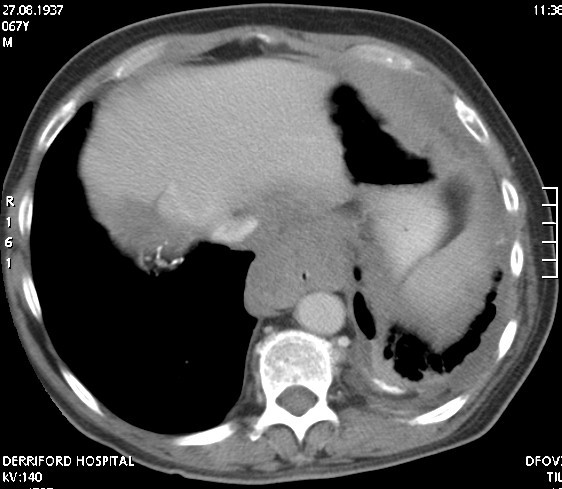
CT scan showing extensive mesothelioma of the chest wall and esophagus.

68-year-old gentleman diagnosed with epitheliod type MM involving the right pleura in January 2005. He developed dysphagia and weight loss in August 2005 and had an endoscopy, which showed extrinsic compression of the esophagus from 25 to 40 cm (Figure 1). He had a SEMS deployed. At the time due to persistent narrow stent aperture, balloon dilation within the stent was also performed. He was readmitted 1 month later with further dysphagia. A repeat endoscopy revealed a food bolus obstruction within a widely patent stent, which was removed. He survived for a further 1 month with no further SEM complications.

## Discussion

Technically, the use of SEMS in the setting of malignant pleural mesothelioma presents several unique challenges. Firstly because there is usually infiltration of long segments of the oesophagus, long stents and occasionally multiple SEMS may be required. Secondly because the oesophagus is infiltrated externally and becomes contiguous with a large tumour mass throughout the mediastinum, esophageal expansion during stent deployment is limited. We have usually attempted to treat this by balloon dilatation of the stent after it has been deployed.

Finally because the underlying disease is progressive, further stenting of previously unaffected section of the esophagus through patent SEMs may be required.

Dysphagia is a rare but recognized complication of malignant mesothelioma. It is usually a very late feature and is usually a terminal event. With more aggressive treatment for malignant mesothelioma, and patients living longer with the diagnosis, this complication may become more common. The average length of survival after diagnosis with malignant mesothelioma in symptomatic patients is 14.7 months [5,6]. The group of patients in this report lived for 6, 8, and 2 months after stent placement. They had a good quality of life and few stent related complications. After stent insertion all 3 patients gained weight indicating that rapid weight loss was not necessarily due to metastatic dissemination of tumour but may in part be due to local effects.

In conclusion, this report shows that active treatment of dysphagia in patients with malignant mesothelioma is beneficial to patients even in the presence of disseminated disease and should therefore be considered in all cases.

## Authors' contributions

EA reviewed case notes and wrote the manuscript

JR performed the operative procedures
